# Translation and psychometric testing of the simplified version of the health-related diet and exercise self-efficacy scale in China

**DOI:** 10.1186/s12955-022-02037-2

**Published:** 2022-08-27

**Authors:** Xia Mao, Xuefang Zeng, Qinyi Zhong, Jia Guo

**Affiliations:** 1 School of Nursing, Yueyang Vocational Technical College, Yueyang, 414000 Hunan China; 2 Xiangya School of Nursing, Central South University, 172 Tongzipo Road, Changsha, 414000 Hunan Province China

**Keywords:** Diet, Exercise, Self-efficacy, Reliability, Validity

## Abstract

**Background:**

Measuring health-related diet and exercise self-efficacy is an important first step in improving healthy behaviors and health outcomes. However, we did not find a self-efficacy measurement in Chinese that is specifically targeted at diet and exercise self-efficacy among healthy adults.

**Aim:**

The present study aimed to translate the Health-Related Diet and Exercise Self-Efficacy Scale -simplified version into Mandarin Chinese (HRDESES) and evaluate its reliability and validity in Chinese healthy adults.

**Methods:**

The HRDESES was translated and adapted to the Chinese context, with a good content validity of 0.86 among seven experts. The survey was then carried out in 216 adults in Hunan, China. Testing of the reliability included internal consistency reliability and test-retest reliability, while validity included content validity, construct validity, and criterion validity.

**Results:**

The Cronbach's α of the HRDESES was 0.87 for the total scale, 0.86 for the diet subscale and 0.91 for the exercise subscale; the McDonald's ω of the HRDESES-SC was 0.85 for the total scale, 0.86 for the diet subscale and 0.91 for the exercise subscale, all demonstrating good internal consistency. The test-retest reliability was 0.88 for the total scale, 0.81 for the diet subscale and 0.82 for the exercise subscale, demonstrating good test-retest reliability. For construct validity, the scale effectively distinguished subjects by age, gender, education, occupation, marital status, and family income, showing good discriminant validity. The confirmatory factor analysis (CFA) supported a two-factor structure of the scale: diet and exercise subscale. It was demonstrated that the HRDESES was highly associated with the General Self-Efficacy Scale and its two subscales, with correlation coefficients ranging from 0.83 to 0.86 (*p* < 0.05), showing high criterion validity.

**Conclusion:**

The HRDESES had good reliability and validity and could be used as a simple and effective tool for assessing the health-related diet and exercise self-efficacy in Chinese healthy adults.

## Introduction

The economic advancements and associated changes in lifestyles have led to the rapid increase in the global incidence of chronic non-communicable diseases (NCDs) [1]. The "Healthy China 2030 Planning Outline" emphasizes implementation of primary prevention among healthy adults to reduce NCDs-related risk factors and to finally reduce and prevent NCDs [2]. Primary prevention highlights taking active measures to reduce the disease-related risk factors before the disease occurs, thus preventing the disease. Previous studies have demonstrated that healthy lifestyles such as healthy diet and regular physical activity are highly effective in preventing NCDs in healthy adults [3]. Such desired healthy lifestyles have been well-documented to be predicted by an important motivational factor—self-efficacy [1–6]

First defined by Bandura based on social cognitive theory [7], the term self-efficacy refers to “belief in one’s capabilities to organize and execute the courses of action required to produce given attainment”. Diet self-efficacy refers to belief in one’s ability to manage a healthy diet, which includes behavior changes through healthier food choice, more consumption of fruit and vegetables, and engagement in healthy cooking at home [8–10]. Exercise self-efficacy refers to belief in one’s ability to maintain a routine exercise schedule to increase both the frequency and intensity of physical activity [11, 12]. Both diet and exercise self-efficacy have been widely reported to be associated with positive and healthy lifestyles behaviors such as healthy diet and regular physical activity [1–6, 12], which further lead to better health outcomes such as improved emotional health [13] and higher quality of life [14].

Considering the essential role diet and exercise self-efficacy played in promoting healthy lifestyles, preventing NCDs, and improving the overall well-being of the individuals, it is important to accurately measure such a concept to guide for further assessment, intervention, and evaluation. One of the most commonly used assessment tools is the health-related diet and exercise self-efficacy scale developed by Sallis and colleagues in 1988 [13]. The scale includes 73 items and have shown favorable psychometric properties among healthy populations in the USA [13]. In 2014, this scale was simplified to 8 items by a USA researcher Manleco CW and has been widely applied in healthy adults in many countries due to fewer items and comparable validity and reliability with the original scale [14, 15]

However, there is no Chinese version of the simplified health-related diet and exercise self-efficacy scale so far and little is known about the diet and exercise self-efficacy in healthy adults in China. To fill in the research gap, we conducted the current study to develop a Chinese version of the simplified health-related diet and exercise self-efficacy scale and to run psychometric testing on this scale among the Chinese healthy adults.

## Materials and methods

### The simplified Chinese version of the health-related diet and exercise self-efficacy scale

We obtained the approval for translation from the original developer of the scale. Based on Brislin’s (1986) adapted translation model [16],the English version was translated into Mandarin Chinese in the following three steps.

*Step 1: Forward translation* Two of the researchers (CG, AE), both were nurses with master’s degree, completed the forward translation of the instrument independently to reduce potential bias of each forward translator. Both researchers spoke Chinese as their native language and were proficient in English. A meeting with an additional clinical expert who is bilingual in Chinese and English was then held to resolve any discrepancies and seek agreement and to reconcile the two forward translations into a single forward translation. Finally, the 1st simplified Chinese version of the Health-Related Diet and Exercise Self-Efficacy Scale was acquired after consultation, discussion and revisions.

*Step 2: Back translation* The preliminary The Health-Related Diet and Exercise Self-Efficacy Scale was translated back into English by a professional translator who spoke English as native language and was proficient in Chinese. The translator was completely blinded to the original version of the instrument. Using the Delphi consultation method, the original English version and the translated version of The Health-Related Diet and Exercise Self-Efficacy Scale were emailed to 2 experts (one is Professor Catherine Waters, another one is a professor of nursing) to compare the backtranslation with the original version to ensure conceptual equivalence between the two versions. Any ambiguities and discrepancies between the two versions were discussed and resolved, leading to some revisions. The backtranslation was then sent to the developer of the original scale for discussion of further revision, leading to the 2^nd^ version of The Health-Related Diet and Exercise Self-Efficacy Scale.

*Step 3: Content validity* The 2nd version of The Health-Related Diet and Exercise Self-Efficacy Scale was then reviewed by an expert committee of 7 clinical nurses for content validity to identify any conceptually or culturally inappropriate items. Content validity was assessed in two aspects: relevance and intelligibility to evaluate experts’ agreement. Each item was rated on a four-point Likert scale from 1 = not relevant to 4 = highly relevant. A content validity index (CVI) at the item level (I-CVI) was developed by dividing the rating of either 3 or 4 by the number of experts [15]. Items with low ratings were also discussed among the experts and reviewed by the research group. Items with I-CVI < 0.78 were further revised accordingly [15]. After several rounds of discussion and revision, the I-CVI of each item was above 0.80, with CVI for the total scale reaching 0.86, indicating good content validity.

### Participants

In order to get a diverse sample that includes populations from various socio-economic backgrounds, we recruit participants from the following three different research sites located in the Yueyang City of Hunan Province: teachers and students from the Yueyang Vocational Technical College, workers from the Yueyang Baling Petrochemical Company, and farmers from the Shiying village, Huarong County of Yueyang City. The inclusion criteria were the following: 1) being ≥ 18 years of age; 2) able to read and communicate; 3) voluntary to participate in this study. The exclusion criteria were: 1) currently hospitalized and receiving medical treatment; 2) with substantial physiological defects or major diseases, which prevented them from engaging in daily works, studies, and living independently; and 3) with cognitive impairment.

The sample size was estimated according to the requirement of 10–25 participants for each item to conduct factor analysis, that is 80–200 participants for this study [17]. Considering a rejection or loss-to-follow-up rate of 10%, our final N of 220 satisfies the sample size requirement.

### Procedure

The study protocol was approved by the Ethics Committee of Xiangya School of Nursing, Central South University (No. 2018028). The recruitment procedure was conducted from May 2020 to August 2020 among 216 healthy adults. After obtaining the consent of the relevant persons in charge of the three research sites, the research team implemented the participant recruitment by putting up posters on each of the research sites through the College Youth League Committee of the Yueyang Vocational Technical College, the Labor Union of the Yueyang Baling Petrochemical Company, and the Village Committee of the Shiying village, respectively. The research team trained three research assistants, who are responsible for the participant recruitment in the three research sites. For potential participants, the research assistant will confirm whether they meet the inclusion criteria, and inform the purpose, significance, and content of this study, as well as information confidentiality and potential risks and benefits. Written informed consent was obtained from each participant before the survey and all information was kept strictly confidential. Participants were invited to complete the survey in a quiet room at the research site. The questionnaire includes a self-report demographical information sheet, the Simplified Chinese version of the Health-Related Diet and Exercise Self-Efficacy Scale, and the Chinese version of the General Self-Efficacy Scale. In addition, 20 subjects were randomly selected to perform the test again two weeks later to evaluate the test-retest validity. All participants were reimbursed with some small gifts such as a towel or soap (RMB ¥ 5) in return for their participation.

### Instruments

The Simplified Chinese version of the Health-Related Diet and Exercise Self-Efficacy Scale included 8 items under 2 dimensions: diet subscale and exercise subscale. Response options are rated on a 5-point scale: 0 (I’m not sure), 1 (mostly I cannot), 2 (don’t know), 3 (mostly I can), or 4 (I’m sure I can). The total score ranges from 0 to 32, with a higher score indicating higher levels of health-related diet and exercise self-efficacy. The original simplified English version has been applied in the United States and has been proven to have good reliability and validity [14]. In the present study, the Simplified Chinese version of the Health-Related Diet and Exercise Self-Efficacy Scale showed good internal consistency with a Cronbach's α of 0.87 for the total scale, 0.86 for the diet subscale and 0.91 for the exercise subscale.

*General Self-Efficacy Scale.* The General Self-Efficacy Scale was used for testing criterion validity of the Health-Related Diet and Exercise Self-Efficacy Scale. The Chinese version of General Self-Efficacy Scale was translated and revised by Wang et al*.* [18] in 2001. It included 10 items, each scored on a 4-level Likert scale from 1 (never) to 4 (always). The total score ranges from 10 to 40, with a higher score indicating a higher level of self-efficacy. A recent study by Zeng et al*.* [19, 20] showed a two-factor structure of the General Self-Efficacy Scale: action self-efficacy and coping self-efficacy. In the present study, the General Self-Efficacy Scale showed good internal consistency with a Cronbach′s α of 0.82 for the total scale, 0.79 for the action self-efficacy subscale, and 0.81 for the coping self-efficacy subscale.

### Statistical analysis

Statistical Package for Social Science (SPSS Inc., Chicago, IL, USA) Version 18.0 for Windows and Mplus 8.0 software were used for all data analysis. The data in this study had a normal distribution. Means ± standard divisions were used for the description of demographic characteristics. Analysis of reliability and validity were described as following:

#### Reliability

Reliability indicates the consistency and stability of scale and includes internal consistency and test-retest reliability [21]. Internal consistency was tested by calculating Cronbach’s α, with a recommended level of 0.80 or above indicating good internal consistency, and a level of at least 0.60 considered as acceptable. Although Cronbach’s α has been the most widely used measure of reliability, recent studies have suggested that McDonald's Omega’s coefficient (ω) should also be added as it represents a more accurate indicator of reliability when assumptions regarding tau equivalence are not met [22, 23]. So, in this study, we calculated both Cronbach’s α and McDonald's ω for internal consistency reliability. Test-retest reliability refers to the stability of the scale [21] and was calculated in a subsample of 20 subjects who were surveyed again 2 weeks later at the same site by the same investigators. Intraclass correlation coefficient (ICC) was calculated for the total score, with a commonly-accepted level of 0.8 considered as acceptable [21].

#### Validity

Validity indicates the authenticity and accuracy of the scale [18] and includes construct validity and criterial validity. Construct validity further includes discriminant validity and confirmatory factor analysis (CFA). Discriminant validity uses inter-group differences to estimate whether the scale could discriminate between different groups of the population by analysis of variances [24]. Previous studies have shown that populations of different ages, sex, education, occupation, marital status, and family income have different self-efficacy levels [25]. CFA validates the capability of the pre-defined factor model in fitting the actual data [26]. Criterion validity refers to the correlation of the new scale with a golden standard scale that measures the same concept. In this study, the General Self-Efficacy Scale and its two subscales were used as the criterion for the evaluation to calculate the Pearson’s correlations with the HRDESES-SC, with a recommended level of 0.7 and above [27].

## Results

### Sample characteristics

We retrieved 216 valid questionnaires among a total of 220 questionnaires distributed, with an effective rate of 98.18%. Of the included subjects, 80 were males (37.0%) and 136 were females (63.0%) and their ages range from 18 to 73 years (37.18 ± 15.30 years). Most of the participants were married (59.7%), had an education level of middle school and above (84.3%), and had a monthly income between 1001 and 5000(73.6%). For occupation, the largest proportion was workers (44.9%), followed by students (28.2%) and farmers (26.9%). The detailed demographic characteristics of the subjects are shown in Table 1.

**Table 1:** Demographic characteristics of the subjects in this study (n = 216)

Characteristics	Group	n	Percentage (%)
Sex	Male	80	37.0
	Female	136	63.0
Educational level	Primary school	34	15.7
	Junior middle school	44	20.4
	Senior middle school	33	15.3
	Junior college	72	33.3
	College or higher	33	15.3
Occupation	Students	61	28.2
	Civil servants (teachers or workers)	97	44.9
	Farmers	58	26.9
Marital status	Unmarried	80	37.0
	Married	129	59.7
	Devoiced	3	1.4
	Widowed or others	4	1.9
	≤ 1000	31	14.4
	1001 ~ 3000	70	32.4
	3001 ~ 5000	89	41.2
	> 5000	26	12.0
Smoking status	Never	160	74.1
	Occasionally	31	14.4
	Always	25	11.6
Alcohol drinking status	Never	128	59.3
	Occasionally	75	34.7
	Always	13	6.0

### Reliability

The Cronbach's α of the HRDESES-SC was 0.87 for the total scale, 0.86 for the diet subscale and 0.91 for the exercise subscale. The McDonald's ω of the HRDESES-SC was 0.85 for the total scale, 0.86 for the diet subscale and 0.91 for the exercise subscale. All these results demonstrated good internal consistency of the HRDESES-SC. The test-retest reliability was 0.88 for the total scale, 0.81 for the diet subscale and 0.82 for the exercise subscale, demonstrating good test-retest reliability.

### Validity

#### Discriminant validity

As shown in Table 2, the score of the HRDESES-SC showed significant differences by gender, educational level, marital status, occupation, and family per-capital income. Males had higher HRDESES-SC scores than females. The HRDESES-SC score also increased with each increasing educational level. Those who were married had higher HRDESES-SC scores than other groups not in marriage. For occupation, the HRDESES-SC score was highest among workers, followed by students, and then farmers. For family income, the HRDESES-SC score was highest in the 30001-5000 group, and lowest in the > 5000 group. All these results indicate the HRDESES-SC had good discriminant validity to distinguish people from various demographical backgrounds.

**Table 2:** The score of the HRDESES-SC by demographics

Characteristics	Score of HRDESES-SC	F/t	*P*
Age		1.350	0.261
18–44	5.02 ± 3.73		
45–59	5.98 ± 2.76		
60–74	5.27 ± 3.70		
Sex		5.450	< .001
Male	6.98 ± 3.86		
Female	4.26 ± 2.90		
Educational level		8.301	< .001
Primary school or lower	6.35 ± 2.40		
Junior middle school	6.07 ± 3.84		
Senior middle school	3.09 ± 3.68		
Junior college	4.47 ± 2.61		
College or higher	6.97 ± 4.27		
Marital status		10.621	< .001
Unmarried	3.74 ± 2.69		
Married	6.31 ± 3.70		
Devoiced	3.67 ± 0.58		
Widowed or others	3.25 ± 2.22		
Occupation		26.676	< .001
Students	6.47 ± 2.45		
Civil servants	9.84 ± 3.33		
Farmers	3.38 ± 3.39		
Family per-capita income (Yuan/month)		5.780	0.001
< 1000	5.47 ± 3,96		
1001 ~ 3000	5.40 ± 3.20		
3001 ~ 5000	6.42 ± 2.99		
> 5000	2.81 ± 2.40		

*CFA* Results of CFA analysis generally supported the a priori specified two-factor structure (see Table 3). The goodness of fit of the two-factor structural model was higher than a single-factor structural model, and the modified two-factor structure showed the best goodness of fit. The relative chi-squares (v2/df) were lower than 3 (2.96), the values for CFI and TLI were close to 1.0 (0.967/0.949), the RMSEAs were between 0.08 and 1.00 (0.095), showing the goodness-of-fit for the data. All these results support for a two-factor structure by CFA.

**Table 3:** Fit index of the model

Model	χ^2^	df	χ^2^/df	CFI	TLI	RMSEA
Single-factor model	383.12	20	19.17	0.659	0.522	0.290
Two-factor model	67.82	19	3.57	0.954	0.932	0.109

#### Criterion validity

The score of the HRDESES-SC was highly and positively associated with the General Self-Efficacy Scale and its two subscales, with a correlation coefficient of 0.86 (*p* < 0.05) for the total score of the General Self-Efficacy Scale, 0.85 (*p* < 0.05) for the subscale of action self-efficacy, and 0.83 (*p* < 0.05) for the subscale of coping self-efficacy. All these results demonstrated high criterion validity of the HRDESES-SC.

## Discussion

This study is the first to translate the simplified Health-Related Diet and Exercise Self-Efficacy Scale into Mandarin Chinese (HRDESES-SC) and test its psychometric properties. This scale was strictly translated according to the Brislin translation model. The forward-translation, back-translation, and content validity testing were performed to acquire the final version of the HRDESES-SC. The translation showed satisfactory psychometric properties, including good content validity among experts, high internal consistency and test-retest reliability, good discriminant validity and criterial validity. In addition, the CFA supported a two-factor structure of the HRDESES-SC: exercise subscale and diet subscale.

Compared with the full scale of Health-Related Diet and Exercise Scale, the HRDESES-SC enjoyed advantages due to its ease of administration, minimization of respondent burden, and quick screening in busy occasions with an average completion time of 8 min. Introducing this scale into mainland China could ensure the standardized evaluation of health-related diet and exercise self-efficacy by healthcare providers in China. In general, the HRDESES-SC provides a convenient and reliable tool for evaluating the health-related diet and exercise self-efficacy among Chinese adults, with implications for future development of primary prevention strategies to improve healthy lifestyles and prevent NCDs.

### Reliability

The internal reliability and test-retest reliability were used to evaluate the consistency and cross-time stability of the scale. Cronbach’s alpha coefficients and McDonald's Omega coefficient for the total scale and two subscales exceeded 0.80, indicating high internal consistency reliability of the HRDESES-SC. This finding is comparable to previous observations showing internal consistency greater than 0.80 for the HRDESES-SC scale in non-Chinese samples [13–15]. High test-retest reliability was supported by high ICC of the total score, also showing the stability of the HRDESES-SC in assessing health-related exercise and diet self-efficacy over time. However, test-retest reliability findings must be interpreted with caution because of the small sample size. Future research is warranted to examine test-retest reliability using a larger sample size.

### Validity

The validity of the scale was evaluated by content validity, construct validity, and criterion validity. The content validity index (CVI) for both the total scale and each item all exceeded 0.80, indicating good content validity. The items of the translated Health-Related Diet and Exercise Self-Efficacy Scale could well reflect the diet and exercise self-efficacy, as assessed by the experts. Consistent with previous studies [25], The Health-Related Diet and Exercise Self-Efficacy Scale score showed significant differences among people with various demographics such as sex, education, occupation, marital status, and family income, indicating that the scale had good discrimination capability.

Regarding the factor structure, the Confirmatory Factor Analysis supported a two-factor structure of the Health-Related Diet and Exercise Self-Efficacy Scale: exercise subscale and diet subscale with generally good model fit indices. These findings provide further support to the original factor analysis of both the full scale of Health-Related Diet and Exercise Scale and its simplified version. These results also demonstrate the robustness of the factor structure of the Health-Related Diet and Exercise Scale even with shorter item numbers. The two-factor structure is also in accordance with our theoretical hypothesis that the Health-Related Diet and Exercise Self-Efficacy Scale covers both exercise and diet aspects.

Criterion validity refers to the correlation between a scale with a gold standard scale. However, there is currently no gold standard for health-related diet and exercise self-efficacy in China. For this study, we used the most commonly used and widely accepted General Self-Efficacy Scale as gold standard. Our findings showed high correlation between the Health-Related Diet and Exercise Self-Efficacy Scale and the General Self-Efficacy Scale and its two subscales, indicating good criterial validity. Self-efficacy is a broad term that refers to belief in one’s ability to perform various actions to realize a goal and may cover various aspects. The high correlation between the Health-Related Diet and Exercise Self-Efficacy Scale and the General Self-Efficacy Scale reflects that the Health-Related Diet and Exercise Self-Efficacy Scale accurately captured the concept and exercise and diet efficacy.

## Limitations and implications

Although this study presented strong empirical evidence for the reliability and validity of the Health-Related Diet and Exercise Self-Efficacy Scale, some possible limitations should also be considered. First, our study subjects were healthy adults recruited from Hunan Province of China and may not represent other populations, such as people with chronic conditions, or people from other areas, such as other parts of China. Future research may consider using conducting national-level study to get a more representative sample. Second, the relatively small sample size of 20 used in the test-retest reliability assessment may lead to potential bias and future research may benefit from recruiting a larger retest sample to get a more robust ICC.

Despite these limitations, our study still provides important clinical and research implications. The 8-item Health-Related Diet and Exercise Self-Efficacy Scale provides a short, self-management, reliable and effective tool that can be used to screen health-related diet and exercise self-efficacy in the non-ill adult population in China. The scale can be used as an effective screening tool to understand people’s diet and exercise self-efficacy so as to guide for the development of targeted health education and health promotion activities related to the prevention of chronic diseases in the general population in the community. In addition, the scale can also be used as an evaluation tool to assess the intervention effects of such health promotion programs.

## Conclusions

As incidences and prevalence of chronic NCDs have been gradually increasing in China, improving diet and exercise self-efficacy is of great importance for early prevention of such diseases. The Chinese version of the Health-Related Diet and Exercise Self-Efficacy Scale has good reliability and validity construct validity to evaluate the diet and exercise self-efficacy among healthy adults in China. The scale can be used as an effective tool to guide for future health promotion programs as well as an evaluation tool to assess intervention effects.

## Data Availability

All research data is available upon reasonable request.
